# Cathepsin B/X is secreted by *Echinometra lucunter* sea urchin spines, a structure rich in granular cells and toxins

**DOI:** 10.1186/1678-9199-19-33

**Published:** 2013-12-16

**Authors:** Juliana Mozer Sciani, Marta Maria Antoniazzi, Adriana da Costa Neves, Daniel Carvalho Pimenta

**Affiliations:** 1 Laboratory of Biochemistry and Biophysics, Butantan Institute, São Paulo, São Paulo State, Brazil; 2 Center of Marine Biology, University of São Paulo (USP), São Sebastião, São Paulo State, Brazil; 3 Laboratory of Cell Biology, Butantan Institute, São Paulo, São Paulo State, Brazil

**Keywords:** *Echinometra lucunter*, Spines, Cathepsin, Proteolysis

## Abstract

**Background:**

*Echinometra lucunter* is a common American sea urchin responsible for the majority of the marine accidents in Brazil. Although not lethal, these accidents are reported to be extremely painful. Recently, our group described the presence of toxins in its spines that contribute to the pathological reactions. Additionally, we have observed that the *E. lucunter* spines can regenerate when broken. In the present work we evaluated the enzymatic activities of sea urchin spine extracts in order to identify an enzyme that could contribute not only to the toxicity, but also participate in the spine growth and regeneration.

**Results:**

The spine aqueous extract was tested for peptidase activity, with synthetic substrates, in the presence and absence of inhibitors and activators. For proper enzyme classification, the FRET-substrate cleavage pattern, pH-dependency activity and Western-blot analyses were performed. The spine extract was able to cleave Z-R-MCA and Abz-GIVRAK(Dnp)-OH following pre-incubation with DTT, and was inhibited by E-64. Furthermore, the double-peaked pH curve (5 and 7) and the cleavage site proportion (4:6, R↓A:A↓K) indicate the presence of both mono and dicarboxypeptidase activities. Moreover, in Western-blot analysis, the spine extract was positive for anti-cathepsin B antibody.

**Conclusions:**

*E. lucunter* spines extracts presented a cysteine peptidase activity that was identified as cathepsin B/X that would participate in the remodeling and growth processes of the spine, as well as in the inflammatory response to the accident.

## Background

Cathepsin cysteine peptidases belong to the C1 cysteine peptidase family of the CA clan, which is also known as the papain family, and comprise a large number of enzymes from both prokaryotes and eukaryotes [1]. Two major cathepsin groups have been described based on their tissue distribution: the first group is composed of ubiquitously expressed members, including cathepsin B, C, F, H, L, O and X, while the second group consists of cathepsin J, K, S and W which have shown restricted expression in certain tissues. Moreover, cathepsins can be divided into three major subgroups based on their sequence homology and specific amino acid motifs; these include the cathepsin B-like, cathepsin L-like and cathepsin F-like genes. Cathepsins are synthesized as inactive precursors and become activated after proteolytic removal of an N-terminal propeptide [2-4].

These enzymes constitute major components of the lysosomal proteolytic system responsible for protein degradation and turnover, playing an important role in maintaining homeostasis in organisms. Movement of lysosomal cathepsins towards the cell membrane or their secretion outside the cell may lead to degradation of the extracellular matrix. This process is usually pathological and contributes to the development of many serious human diseases such as cancer, arthritis, osteoporosis, Alzheimer's disease, multiple sclerosis, inflammation etc. [3,4].

Cathepsin X (EC 3.4.18.1, nomenclature according to the NC-IUBMB) – previously known as carboxypeptidase LB, cathepsin IV, cathepsin B2, cathepsin P, cathepsin Y, cathepsin Z, cathepsin Z1, CTSZ g.p., cysteine-type carboxypeptidase or lysosomal carboxypeptidase B – is a carboxypeptidase that preferentially degrades substrates as carboxymonopeptidases. It cleaves substrates containing Arg at the antepenultimate position as carboxydipeptidases, demonstrating a very unusual switching between monopeptidyl and dipeptidyl peptidases, hence its initial name cathepsin B2. Moreover, it exhibits little or no endopeptidase activity, another reason for such assessment [5,6].

Cathepsin X and cathepsin B (EC 3.4.22.1) share several features. The superimposition of cathepsin X (DOI: 10.2210/pdb1deu/pdb) and cathepsin B (DOI: 10.2210/pdb1csb/pdb) structures indicates that His^23^ of cathepsin X occupies a region in space which partially overlaps His^110^ of cathepsin B, a residue considered to be responsible for the exopeptidase activity of the latter enzyme. Cathepsin B is known to hydrolyze substrates through a dipeptidyl carboxypeptidase pathway, and also displays a lower but significant endopeptidase activity [5,7].

Cathepsins are involved in the digestion of yolk proteins in oocytes, fertilized eggs and the yolk sac. Moreover, cathepsins are also normally found in non-ovarian tissues of fish where they can be involved in cellular degradation of proteins in other process including death, spawning or starvation; as well as having bacteriolytic and defensive roles [8].

*Echinometra lucunter* is a sea urchin commonly found in the Brazilian shoreline whose spines are composed by calcium and/or magnesium carbonate. Accidents with humans are frequent, usually involving several stings on feet or hands. There are cells within the calcified matrix that are able to secrete toxins that may take part into the accident, complementing the mechanical trauma caused by the spines [9-12]. These cells may also be involved in the regeneration process, which is a known mechanism triggered whenever a spine breaks [13].

In this work we identified the presence of a cysteine peptidase activity in the aqueous extract of *E. lucunter* spines. By analyzing the enzyme kinetic parameters, antibody (Ab) recognition pattern and histological observations, we classified this enzyme as a cathepsin B/X that could be involved in the regeneration process of the broken spines, as well as in the defense of the sea urchin.

## Methods

### Reagents

All the employed reagents were purchased from Sigma Co. (St. Louis, USA), unless otherwise stated.

### Sea urchin spine extract

Specimens of *E. lucunter* were collected in São Paulo, Brazil (23°49’53”S; 45°31’18”O), under license number 13852-1 from the Brazilian Institute of Environment and Renewable Natural Resources (IBAMA). Animals were collected without distinction of sex, age or size. Spines were removed by cutting the connective tissue with a scissor, then they were washed very quickly with distillated water to remove sand and algae, and immediately immerged in ammonium acetate (100 mM) for 24 hours, at 4°C. After that, the solution was centrifuged at 9500 g for seven minutes, and the supernatant was used in the experiments.

The protein content was assessed by reactivity to Bradford reagent (compared to albumin curve as standard), to kinetic assays and western blotting experiments.

Analysis and fractioning by HPLC were performed in a gel-filtration column (TSKgel® Super SW2000, 46 × 300 mm, TOSOH Bioscience, Japan) and the contents were eluted with a solution of 1 M NaHPO_4_ and 1 M NaCl, pH 6.7, under a constant flow of 0.3 mL.min^-1^. Fractions were collected at one-minute intervals and were all assayed by enzymatic effect using FRET substrates (described in “Kinetic assays” section).

### Kinetic assays

Z-R-MCA (carbobenzoxy-L-arginine-7-amino-4-methylcoumarin) was purchased from Sigma and the FRET substrates were a kind gift of Aminotech P&D Ltda (Brazil).

Hydrolysis of Z-R-MCA substrate was monitored by the fluorescence emission (λ_ex_330 nm and λ_em_430 nm) at 30°C in 100 mM ammonium acetate (CH_3_COONH_4_), pH 7.4, in a SpectraMax® Gemini XPS spectrofluorimeter (Molecular Devices, USA). The enzyme solution was added to the 96-well microplate containing the substrate solution and the increase in fluorescence over time was continuously recorded for up to 40 minutes. Alternatively, the enzyme solution was preincubated with 2 mM DTT for ten minutes, at room temperature. PMSF (1 mM), aprotinin (0.3 μM), EDTA (1 mM) and E-64 (9 μM) were individually added to the DTT-activated enzyme solution, prior to the substrate addiction for catalytic mechanism assessment.

The FRET substrates Abz-GIVRAK(Dnp)-OH (Abz: *o*-aminobenzoic acid; Dnp: 2,4-dinitrophenyl) and Abz-GIVRAKQ-EDDnp [EDDnp; N-(2,4-dinitrophenyl)-ethylenediamine] were employed for exo- and endopeptidase activity, respectively. The assay was performed in 100 mM CH_3_COONH_4_, containing 200 mM NaCl, pH 4.5 and the fluorescence was measured at λ_ex_ 320 nm λ_em_ = 420 nm in a SpectraMax® M2 spectrofluorimeter (Molecular Devices, USA), in a 1 cm path-length cuvette. The increase in fluorescence with time was continuously recorded for up to ten minutes. The aqueous extract was also assayed over FITC-conjugated casein, as described by Twining [14].

The pH-dependent activity of the enzyme over the hydrolysis of Z-R-MCA was determined by assaying the enzyme activity (V_max_) in CH_3_COONH_4_ buffer solutions ranging from pH 2.0 to 8.0 (1 unit interval).

Active site titration was performed with E-64, according to Salvesen and Nagase [15], using Abz-GIVRAK(Dnp)-OH substrate. Cleavage site identification was achieved by incubation of spine extract and Abz-GIVRAK(Dnp)-OH substrate and then manually separating the hydrolysis products by RP-HPLC, followed by mass spectrometry analyses.

Non-linear regression data fitting was performed and the kinetic parameters were calculated according to Wilkinson [16], using the Grafit® software (UK).

### Western blotting

SDS-PAGE (10%) was used to separate proteins of the spine extract (10 μg, determined by Bradford assay), according to the method described by Laemmli [17], and subsequently transferred onto nitrocellulose membranes. Briefly, membranes were blocked for one hour with shaking at 4°C in 0.3% serum albumin in Tris-buffered saline with Tween-20 (TBS-T). Membranes were incubated with primary antibody, a mouse monoclonal anti-cathepsin B or anti-cathepsin K, 1:250 (Sigma Co., USA) for ten minutes, in SNAP i.d.® Protein Detection System (Millipore, USA). Membranes were washed three times for ten minutes each with TBS-T. Horseradish-peroxidase-conjugated secondary antibody (1:333) was added for ten minutes, followed by a wash in TBS-T. Protein signals were detected using enhanced chemiluminescence Western blotting detection reagents (GE Healthcare, UK).

### Histology and immunohistochemistry

For such analyses, spines were removed *in loco* and immediately fixed by immersion in Karnovsky solution [18]. After 48 hours, spines were decalcified in a solution of 4% EDTA, pH 7.2, under constant agitation for 4 to 6 hours, dehydrated in ethanol series (70 to 100%) and embedded in glycol methacrylate (Leica Microsystems, Germany). Transversal 2-μm sections were obtained with a Microm HM340 E® (Thermo Fisher Scientific, USA) microtome. Sections were stained with toluidine blue-basic fuchsin.

Immunohistochemical reactions were performed using monoclonal anti-cathepsin B produced in mouse (Sigma Aldrich, MO). Three-micrometer sections of spine were deparaffinized, rehydrated, and incubated in 6% aqueous hydrogen peroxide in methanol (1:1) for 30 minutes to quench endogenous peroxidase activity. The slides were not submitted to antigen retrieval treatment. The sections were incubated with anti-B cathepsin at 8 μg/mL for two hours at room temperature. ADVANCE® HRP system (Dako, USA) was used to detect cathepsin antibodies. The specimens were lightly counterstained with Mayer’s hematoxylin, dehydrated, and mounted onto glass coverslips and xylene-based mounting medium. No immune serum was used as negative control.

Photomicrographs were obtained in an Olympus BX51® microscope coupled to an Olympus QColor 5® camera (Tokyo, Japan), using Image-Pro® Express software (Media Cybernetics, USA) for image capture.

## Results

**Figure 1:**
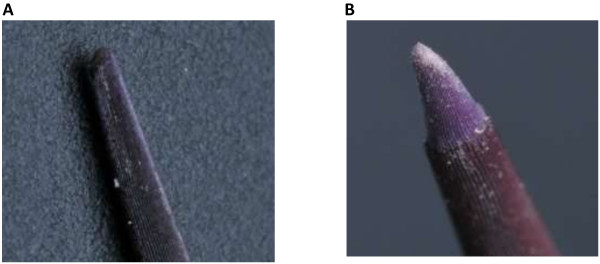
**Photography of the *Echinometra lucunter *** **spine tip: (A) intact spine; (B) regeneration process.**

As described for other animals, the presence of peptidases is important to the growth and regeneration of eggs, shells and exosqueleton [19]. The regeneration process was observed in the tip of *Echinometra lucunter* spines, as showed in Figure 1. Then, a screening for proteolytic activities was performed.

**Figure 2:**
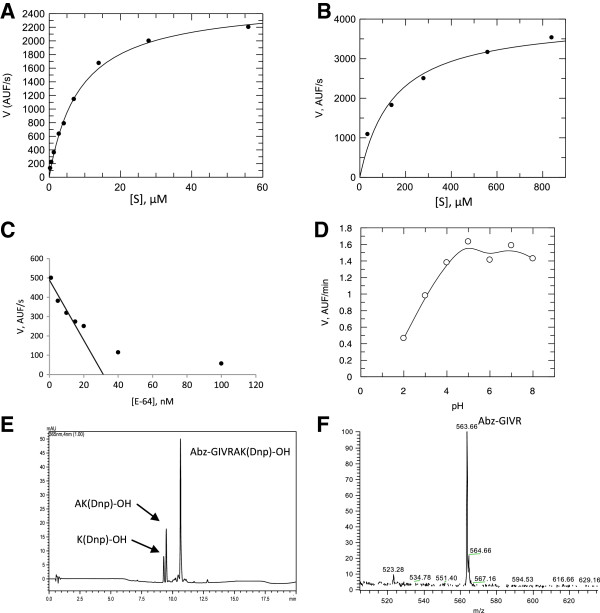
**Enzymatic characterization of the spine extract.** Kinetic data of velocity over concentration of substrate, after the incubation of spine aqueous extract (12 μg) with **(A**) Z-R-MCA and **(B)** Abz-GIVRAK(Dnp)-OH. **(C)** Enzyme titration using E-64 inhibitor, performed over Abz-GIVRAK(Dnp)-OH substrate. **(D)** Determination of pH for optimum activity of spine aqueous extract, over Abz-GIVRAK(Dnp)-OH substrate. **(E)** HPLC profile, in λ = 365 nm, of the products of complete hydrolysis of the Abz-GIVRAK(Dnp)-OH substrate by spine aqueous extract. **(F)** Mass spectrometry analysis of the products of Abz-GIVRAK(Dnp)-OH hydrolysis by spine aqueous extract.

When the spine extract (12 μg) was assayed over Z-R-MCA substrate, no proteolytic activity was observed. However, when the spine extract was preincubated with DTT, an activity was observed, as shown in Figure 2 – A and Table 1. E-64 was the only one that could inhibit this activity, indicating the presence of cysteine peptidases.

**Table 1:** **Kinetic parameter for the hydrolyses of FRET and fluorogenic peptides by *E. lucunter *** **aqueous extract cathepsin B/X**

**Substrate**		**K**_**M **_**(μM)**	**k**_**cat **_**(s**^**-1**^**)**	**k**_**cat**_**/K**_**M **_**(mM.s)**^**-1**^
Z-R-MCA		147.2 ± 40.0	7.46 ± 0.60	51 ± 4
Abz-GIVR↓AK(Dnp)-OH	(63%)^1^	8.57 ± 0.51	0.29 ± 0.01	34 ± 2
Abz-GIVRA↓K(Dnp)-OH	(37%)			
Abz-GIVRAKQ-EDDnp		ND^2^	ND	ND

^1^As determined by HPLC product peak area.

^2^Not determined due to poor hydrolysis.

In order to better evaluate the nature of the cysteine peptidase present in the spine extract, the specific FRET substrate Abz-GIVRAK(Dnp)-OH was tested (Figure 2 – D, Table 1), as well as its C-terminal blocked analog Abz-GIVRAKQ-EDDnp. When Abz-GIVRAK(Dnp)-OH was used, an important activity was observed (Figure 2 – B, Table 1), while Abz-GIVRAK-EDDnp was virtually resistant to hydrolysis (Table 1), indicating an exopeptidase activity. This spine extract failed to cleave the C-terminal FRET substrate, and neither was able to cleave the FITC-conjugated casein (data not shown), behaving as a carboxypeptidase.

The inhibition of proteolytic activity over Abz-GIVRAK(Dnp)-OH by E-64 is shown in Figure 2 – C. The aqueous spine extract employed throughout this work (corresponding to circa 600 spines; ten animals and 0.12 mg.mL^-1^ protein) contained 30 nM cysteine peptidase, as titrated with E-64. The pH-dependence of this hydrolysis was also analyzed, as presented in Figure 2 – D, which shows two maximums pH: 4.4 and 6.8.

C18-RP-HPLC was performed to isolate the cleavage products over Abz-GIVRAK(Dnp)-OH, as shown in Figure 2 – E, that were identified by MS (Figure 2 – F). Two cleavage sites were identified: after arginine and after alanine.

**Figure 3:**
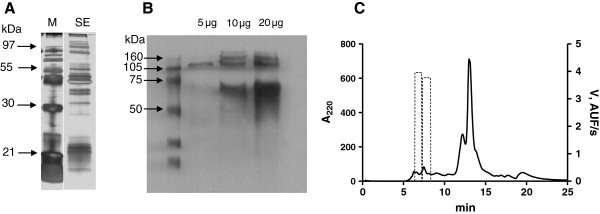
**Proteic characterization of the spine extract. (A)** SDS-PAGE (10%) of aqueous extract of spine. M = molecular mass standard, SE = spine extract. Arrows indicate the molecular masses. **(B)** Western blotting of 5, 10 and 20 μg aqueous extract of spine, incubated with anti-cathepsin B antibody. Left lane, molecular mass standard. **(C)** Chromatogram obtained by gel filtration of spine aqueous extract. Dashed lines indicate the enzymatic activity.

In order to confirm the nature of cathepsin, Western blotting (WB) experiments were performed with anti-cathepsin B antibody and anti-cathepsin K. The rather complex protein composition of the spine extract has been already assessed by SDS-PAGE (Figure 3 – A), so the recognition pattern of WB analysis by anti-cathepsin B was considered specific (Figure 3 – B) [12]. There was no recognition using anti-cathepsin K and when higher exposition of the membrane was employed, unspecific background staining was observed (data not shown).

A gel-filtration separation was performed and all the fractions were assayed for cysteine peptidase activity over Z-R-MCA. One can observe that only the higher molecular mass fractions presented proteolytic activity (Figure 3 – C), in accordance to the WB analysis.

**Figure 4:**
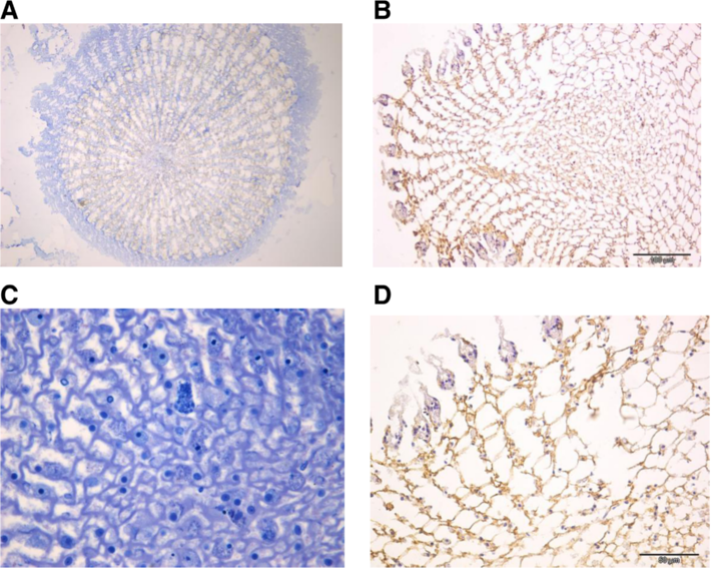
**Immunohistochemical test for anti-cathepsin B antibody was performed in transversal spine sections. (A)** Spine section stained with toluidin-fuchsin. **(B)** Spine section incubated with anti-cathepsin **B**. **(C** and **D)** zoomed images, corresponding to **A** and **B**, respectively. It is possible to observe the positive (brownish staining) along the decalcified matrix **(B)**, in the same location where cells were observed in the sections stained by toluidine-fuchsin **(A)**.

Toluidine blue-fuchsin stained histological sections of the spine (Figure 4 – A) show that the spaces within the calcified matrix are walled by a continuous filamentous structure forming a framework for longitudinal interconnected compartments (or canals) containing several different types of cells, some of them full of cytoplasmic granules. These cells seem to be proportionally more concentrated at the spine tip (data not shown).

Immunohistochemical test for anti-cathepsin B antibody was performed in transversal spine sections. It is possible to observe the positive (brownish staining) along the decalcified matrix (Figure 4 – B), in the same location where cells were observed in the section, stained by toluidine-fuchsin (Figure 4 – A).

## Discussion

The regeneration of the calcified matrix is a known process that has been described for some groups (review by Dubois and Ameye [13]). However, this event still remains unclear at certain points. Spine regeneration is a positive constitutive process taking place in *E. lucunter* as depicted in Figure 1. So, we chose to biochemically investigate this process. Spine tip regeneration must be very important for this animal, since its defense relies mainly on the mechanical trauma inflicted by the spines, which is associated with the inflammatory reaction caused by the molecules present in the spine [12].

A known molecule, frequently associated to matrix remodeling processes, is the proteolytic enzyme cathepsin, particularly the cysteine peptidase cathepsins. The involvement of these proteins in digestion of yolk proteins in oocytes, fertilized eggs and the yolk sac have already been described [20]. Wang *et al*. [21] verified the participation of a cathepsin B in the embryonic and larval development of *Meretrix meretrix*. The activity of the enzyme altered significantly the shell length of the animals, which was shorter when the enzyme was inhibited.

In cods, such enzymes are primarily involved in the proteolytic digestion of the egg vitellogenin but may have an additional defensive role, as has been demonstrated, for example, in the mucus of adult fish [8]. In the yellow-fever mosquito (*Aedes aegypti*), for example, vitellogenic cathepsin B-like protease participates in embryonic degradation of vitellin [22]. In nematodes, this family of enzymes has a role in molting and in cuticle and eggshell remodeling, involving proteases for the degradation of cuticular proteins. Additionally, proteases may also be involved in the processing of proproteins that are subsequently incorporated into the new cuticle [23]. Moreover, its mammal relative, cathepsin K, has been associated to osteoclast in bone remodeling [24].

In this work, we report a cysteine peptidase activity that was detected in *E. lucunter* aqueous spine extract. This cysteine peptidase cathepsin activity, seemly unique (e.g., due to one single enzyme) was termed as cathepsin B/X, for it presents both carboxi mono- and dipeptidyl peptidase activities, and virtually no endopeptidase activity [25]. Moreover, complementary assays (cleavage pattern of specific substrates, DTT activation, active site E-64 inhibition, pH dependence of the activity, molecular mass evaluation and Ab recognition pattern) corroborate such statement. We used the commercial specific FRET substrate Abz-GIVRAK(Dnp)-OH (Aminotech P&D Ltda., Brazil) and obtained kinetic parameters (K_M,_ k_cat_) very similar to those described for cathepsin B, X and cruzain [26]. Its C-terminal blocked analog, Abz-GIVRAKQ-EDDnp, was virtually resistant to the spine extract, as well as the FITC-casein, excluding a cathepsin L activity on the aqueous extract.

Cotrin *et al*. [26] verified the cleavage site of cathepsins B and X with the Abz-GIVRAK(Dnp)-OH: cathepsin B cleaved 100% after arginine residue and cathepsin X cleaved 100% after alanine, the two cleavage sites observed with the spine extract. The K_M_ value observed for cathepsin B by Cotrin *et al*. [26] was 5.9 μM, similar for the spine extract, which was calculated 8.57 μM. Other cleavages observed by the authors were by cathepsin L, after valine (55%) and after arginine (45%). We did not observed cleavage after valine, so we can discard the presence of this type of cathepsin. Moreover, we did not verify an endopeptidase activity, what confirm the absence of cathepsin L.

Although an approximately 6:4 cleavage ratio (carboxydi:carboxymonopeptidyl peptidase activity) could be observed for Abz-GIVRAK(Dnp)-OH after incubation with the aqueous spine extract, this fact could be explained by the switching catalytic mechanism of cathepsin X. It is based on structural data: the crystal structure of cathepsin X suggests that the positively charged imidazolium ring of His^23^ can switch between two conformations. In the conformation observed in the crystal structure, this ring can bind to the carboxyl group of asubstrate P_1_’ residue, which can form an additional hydrogen bond with the hydrogen of NE1 atom of Trp^202^. On the other hand, a modeling study has suggested that the ring of His^23^ can be brought into the position equivalent to the position of His^110^ in cathepsin B structure by simple rotations about the χ_1_ and *χ*_2_ angles. With the His^23^ ring in the cathepsin B-like conformation, cathepsin X can bind the carboxyl group of a substrate P_2_’ residue. Although the binding would not be the same as in cathepsin B, due to the lack of an equivalent to His^111^, both cleavages (mono and di) would occur [5].

Finally, in order to confirm the presence of cathepsin B in the spine extract, we performed a Western blotting assay. Bands clearly visible, for instance, between 60 and 80 kDa, corresponding to some described cathepsins, such as 81.367 kDa cathepsin X from *Ectocarpus siliculosus* (brown alga) [Uniprot D8LCL2]; 59.358 kDa cathepsin B from the same alga [Uniprot D8LG82] and 65.836 kDa cathepsin B from *Trichinella spiralis* (a nematode parasite) [Uniprot E5SL05]*.* The same antibody used in WB was used in immunohistology (Figure 4), and the recognition pattern matched that of the cellular canals (Figure 4).

It has been reported that venomous animals contain protein secretory cells, which secrete venom as a defense mechanism. Moreover, we reported toxic effects of inflammation and pain of this *E. lucunter* spine extract, indicating that molecules (toxins) are being secreted, probably from these cells [12].

## Conclusion

In conclusion, *E. lucunter* spine extracts showed a proteolytic activity that, based on the selected experiments (kinetic assays, Ab recognition pattern and molecular mass assessment), could be classified as cathepsin B/X, due to its particular substrate cleavage pattern. This enzyme ought to participate in the remodeling process and growth of the spine and may behave as a toxin as well.

### Ethics committee approval

The collection and study of *E. lucunter* sea urchins were approved by the Brazilian Institute of Environment and Renewable Natural Resources (IBAMA) under license number 13852-1.

## Competing interests

The authors declare that there are no competing interests.

## Authors’ contributions

JMS, Ph.D. student from the University of São Paulo, collected the sea urchins and carried out experiments of purification, Western blotting and kinetic assays; MMA and ACM performed the histology and immunohistochemistry experiments. DCP designed the work and supervised the conduction of most experiments. All authors contributed to the final manuscript. All authors read and approved the final manuscript.
